# Real‐world use of Cenobamate in pediatric drug‐resistant epilepsy: A European multicenter retrospective study

**DOI:** 10.1002/epi4.70301

**Published:** 2026-07-03

**Authors:** Konstantin L. Makridis, Thomas Bast, Stéphane Auvin, Pavel Krsek, Matyas Ebel, Eva Breuer, Mira Beckhaus, Matúš Bašovský, Pavlína Danhofer, Ondřej Horák, Alessandro Orsini, Gonzalo Alonso Ramos Rivera, Hana Medřická, Steffen Syrbe, Angela M. Kaindl

**Affiliations:** ^1^ Department of Pediatric Neurology Charité – Universitätsmedizin Berlin Berlin Germany; ^2^ Center for Chronically Sick Children Charité – Universitätsmedizin Berlin Berlin Germany; ^3^ Institute of Cell‐ and Neurobiology Charité – Universitätsmedizin Berlin Berlin Germany; ^4^ Section CNS Development and Neurologic Disease, Partner Site Berlin German Center for Child and Adolescent Health (DZKJ) Berlin Germany; ^5^ Epilepsy Center Kork Kehl Germany; ^6^ AP‐HP, Pediatric Neurology Department, Reference Center for Rare Epilepsies, Member of ERN Epicare Hôpital Universitaire Robert Debré Paris France; ^7^ Institut Hospitalo‐Universitaire Robert‐Debré du Cerveau de L'enfant Paris France; ^8^ Université Paris‐Cité, INSERM NeuroDiderot Paris France; ^9^ Institut Universitaire de France (IUF) Paris France; ^10^ Department of Paediatric Neurology, Motol Epilepsy Center, 2nd Faculty of Medicine Charles University and Motol University Hospital, Full Member of the ERN EpiCARE and Epilepsy Research Centre Prague – EpiReC Consortium Prague Czech Republic; ^11^ Epilepsy‐Center Berlin‐Brandenburg Ev. Krankenhaus Königin Elisabeth Herzberge Berlin Germany; ^12^ Department of Child Neurology Thomayer University Hospital Prague Czech Republic; ^13^ Department of Paediatric Neurology Faculty of Medicineof Masaryk University and University Hospital Brno, Brno Epilepsy Center, EpiCARE Brno Czech Republic; ^14^ Pediatric Neurology University Hospital of Pisa, Azienda Ospedaliero Universitaria Pisana Pisa Italy; ^15^ Department of Neurology Bory Hospital Bratislava Slovakia; ^16^ Department of Paediatric Neurology University Hospital Ostrava Ostrava Czech Republic; ^17^ Division of Pediatric Epileptology, Center for Pediatric and Adolescent Medicine, Clinic 1, Medical Faculty of Heidelberg Heidelberg University Heidelberg Germany

**Keywords:** anti‐seizure medication, cenobamate, drug‐resistant epilepsy, epilepsy, pediatric

## Abstract

Pediatric drug‐resistant epilepsy (DRE) remains a significant clinical challenge, with few effective pharmacological options. This European multicenter retrospective study assessed the real‐world efficacy, tolerability, and retention of off‐label cenobamate (CNB) in 108 children and adolescents with DRE (median age: 13.83 years, range 3.9–19.8). Overall, 57/108 patients (52.8%) achieved a ≥50% seizure reduction, including 20/108 (18.5%) who were seizure‐free during the documented interval between visits. The median monthly seizure frequency declined from 30 to 9. Median follow‐up was 581.5 days (IQR 340,8; range 27–1160); 5 patients had follow‐up ≤90 days and 10 had follow‐up <180 days. In sensitivity analyses excluding patients with follow‐up ≤90 days and <180 days, ≥50% responder rates were 54.4% and 56.1%, respectively. Concomitant ASM dose reductions and discontinuations occurred in 44% and 51% of patients, respectively, with the median ASM count declining from 3 to 2. Adverse events were documented in 57.4%, most frequently fatigue, vertigo, and behavioral symptoms. No cases of DRESS were reported. CNB retention at 12 months was 86.8% and was higher in patients without adverse events (93.5% vs. 81.6%, *p* = 0.0076). The initiation dose was most commonly 12.5 mg (0.25 mg/kg/day, IQR 0.12), with titration to a median maintenance dose of 3.57 mg/kg/day (IQR 1.95; range 0.3–10). These findings suggest that CNB is a generally well‐tolerated and clinically effective adjunctive treatment in pediatric DRE, with the potential to reduce polypharmacy and support long‐term adherence. Controlled prospective studies are needed to confirm these results and to establish safe, evidence‐based use of CNB in children.

**Plain Language Summary:**

Many children and adolescents with drug‐resistant epilepsy do not respond to available medications. In this European multicenter study, we evaluated cenobamate, used off label, in 108 young patients with severe epilepsy. More than half had their seizures reduced by at least 50%, and nearly one in five became seizure‐free. Seizure frequency dropped markedly, and many patients could reduce other anti‐seizure medications. Side effects were common but usually mild, and most patients continued treatment long term. These results suggest cenobamate may be a useful option in pediatric drug‐resistant epilepsy, but prospective studies are needed to confirm safety and effectiveness.

**Key points**

- In this large multicenter cohort, cenobamate provided meaningful seizure reduction in refractory pediatric epilepsy, with nearly 20% achieving seizure freedom.
- Adverse events were mostly mild, and 12‐month retention exceeded 85%, supporting good real‐world tolerability.
- Cenobamate allowed reduction of concomitant anti‐seizure medications, suggesting potential to lessen or reduce treatment burden in complex polytherapy.

## 1 INTRODUCTION

Epilepsy is one of the most common chronic neurological conditions in childhood and adolescence. In one third of affected children, seizures persist despite trials of at least two appropriately selected and dosed anti‐seizure medications (ASM), meeting the criteria for drug‐resistant epilepsy (DRE).^1^ DRE is associated with increased mortality, developmental delay, and reduced quality of life.^2^ While non‐pharmacological interventions can offer benefit in selected cases, most children with DRE continue to rely on pharmacological treatment.^3^

Cenobamate (CNB) is a novel ASM with a dual mechanism of action, combining modulation of voltage‐gated sodium channels with positive allosteric effects on GABA_A_ receptors.^4^ In adult patients with focal epilepsy, CNB has demonstrated superior efficacy compared to other third‐generation ASM, with seizure‐freedom rates exceeding those observed in contemporary trials.^5^

Despite the clinical need, CNB is not yet approved for use in children. To date, published experience with CNB in pediatric patients is limited to small retrospective series and early multicenter efforts.^6^, ^7^ These initial reports suggest that CNB may be effective in younger patients with focal epilepsy, but larger studies are needed to evaluate its use more systematically.^8^

In this study, we present data from one of the largest multicenter cohorts of pediatric patients with DRE treated with CNB.

## 2 METHODS

### 2.1 Study design and cohort definition

We conducted a retrospective, multicenter study across European epilepsy centers to assess the off‐label use of CNB in pediatric patients with epilepsy (Charité – Universitätsmedizin Berlin; Epilepsy Center Kork, Universitaire Robert Debré, Paris; Charles University and Motol University Hospital, Prague; Epilepsy‐Center Berlin‐Brandenburg; Thomayer University Hospital, Prague; Brno Epilepsy Center; University Hospital of Pisa; Bory Hospital; University Hospital Ostrava Heidelberg University). Patients were eligible for inclusion if CNB treatment had been initiated prior to the age of 18 years. Clinical data were extracted from electronic and paper‐based medical records using a standardized data collection sheet.

### 2.2 Clinical classification and baseline assessment

Seizure types and epilepsy syndromes were classified according to the International League Against Epilepsy (ILAE) 2025 classification systems. Baseline monthly seizure frequency was estimated retrospectively based on seizure counts documented in the electronic patient record covering the three months preceding CNB initiation.

### 2.3 Outcome measures

The primary outcome was the change in monthly seizure frequency from baseline to the last documented follow‐up, analyzed on an intention‐to‐treat basis. Because seizure counts were retrospectively abstracted and not prospectively standardized, efficacy was additionally summarized using four mutually exclusive categories: seizure freedom, 50%–99% seizure reduction, 1%–49% seizure reduction, and no improvement or worsening. Seizure freedom was defined as the complete absence of clinical seizures for at least the interval between two consecutive clinical assessments (typically ≥3 months). A responder was defined as a patient with a ≥50% reduction in monthly seizure frequency relative to baseline. Treatment retention was analyzed as a secondary outcome and defined as the proportion of patients remaining on CNB therapy over time, assessed using Kaplan–Meier survival analysis from treatment initiation to discontinuation or last follow‐up. Due to the retrospective design, seizure‐type–specific effects and minimum duration of seizure control beyond the inter‐visit interval could not be systematically verified across centers.

### 2.4 Statistical analysis

Descriptive statistics were applied to summarize patient characteristics. Continuous variables were presented as medians with interquartile ranges (IQR), and categorical variables as absolute frequencies and percentages. Group comparisons for categorical variables were performed using Pearson's Chi‐squared or Fisher's exact tests, as appropriate. Continuous variables were compared using non‐parametric tests as appropriate. Missing data were handled by available‐case analysis without imputation, and denominators are reported where relevant. All analyses were performed using R version 4.2.1. This study was approved by the local ethics committee (approval no. EA2/084/18).

## 3 RESULTS

### 3.1 Cohort

A total of 108 pediatric patients with drug‐resistant epilepsy from 11 European centers met the inclusion criteria. Demographic and baseline clinical data are detailed in Table 1. Of these, 51 (47%) were female. Intellectual disability was present in 71% of the cohort. The median age at epilepsy onset was 3 years (IQR 5.8; range 0.1–16). Most patients met ILAE criteria for drug resistance, with a median of 6 prior ASM (IQR 6; range 1–21) excluding baseline ASM. Patients were taking a median of 3 concomitant ASM at baseline (IQR 2; range 1–6). 73/108 (67.6%) were on ≥3 concomitant ASM.

**TABLE 1:** Patient baseline characteristics. If more than one etiological category applied (e.g., genetically determined structural epilepsy), each etiology was recorded separately. As a result, the total number of etiological entries exceeds the number of individual patients.

Age; median (IQR, range)	
Current age	15.2 (11.9–17.4; 3.9–19.8)
Epilepsy onset	3 (0.7–6.5; 0.1–16)
Treatment start	13.8 (11.5–15.6; 3.6–18)
Female, *n* (%)	51 (47.2)
Seizure type, *n* (%)	
Focal aware (motor/nonmotor)	55 (50.9)
Focal impaired awareness (motor/nonmotor)	62 (57.4)
Focal to bilateral tonic–clonic	34 (31.5)
Generalized onset (motor/nonmotor)	45 (41.7)
Etiology; *n*	
Structural	61
Genetic	30
Immune	7
Infectious	5
Unknown	29
Epilepsy type, *n* (%)	
Unifocal	35 (32.7)
Multifocal	38 (35.5)
Combined generalized and focal	34 (31.8)
Intellectual disability, *n* (%)	77 (71.3)
Monthly baseline seizure frequency	
Median (IQR, range)	30 (91.25, 1–750)
<20 seizures per month, *n* (%)	43 (39.8)
≥20 seizures per month, *n* (%)	65 (60.2)
ASM; median (IQR, range)	
Concomitant at baseline	3 (2, 1–6)
Failed (excluding baseline)	6 (6, 1–21)
Other prior therapies, *n* (%)	
Epilepsy surgery	26 (24.1)
VNS	26 (24.1)
Ketogenic diet	26 (24.1)
Immunomodulation	15 (13.9)

Seizure types at baseline were predominantly focal. Specifically, 62 patients (57.4%) had focal impaired consciousness seizures (FIC), 55 patients (50.9%) had focal preserved consciousness seizures (PBC), and 31.5% (*n* = 34) had focal to bilateral tonic–clonic seizures. 45 patients (41.7%) reported generalized seizures. EEG data were available in 107 patients and revealed a focal epileptiform focus in 35 (32.7%), multifocal epileptiform discharges in 38 (35.5%), and combined generalized/focal epileptiform activity in 34 (31.8%).

Most commonly patients had structural causes (*n* = 61), followed by monogenetic etiologies (*n* = 30). 7 patients had immune causes; 29 patients had unknown causes. Prior non‐pharmacological treatments were common: 24.1% of patients (*n* = 26) each had undergone epilepsy surgery, vagal nerve stimulation (VNS) or a trial of ketogenic diet. 15 patients (13.9%) had received immunomodulatory treatments with cortisol or immunoglobulins.

### 3.2 Treatment

CNB treatment was initiated at a median age of 13.8 years (IQR 4,1; range 3.6–18), corresponding to a median epilepsy duration of 9 years (IQR 7). The initial CNB dose was predominantly 12.5 mg, administered at a median body weight of 47 kg (IQR 24,5; range 12.8–105), reflecting a median starting dose of 0.25 mg/kg/day (IQR 0.12; range 0.12–0.98). In 10 patients, treatment commenced with a lower starting dose of 6.25 mg. Titration led to a median maintenance dose of 150 mg/day (IQR 100), equivalent to 3.57 mg/kg/day (IQR 1,95; range 0.3–10).

### 3.3 Retention

Retention Median follow‐up was 581.5 days (IQR 340,8; range 27–1160). Five patients had follow‐up ≤90 days and 10 had follow‐up <180 days. The Kaplan–Meier estimated probability of treatment retention on CNB was 91.7% (95% CI: 86.6–97.0) at 6 months and 86.8% (95% CI: 80.5–93.5) at 12 months (Figure S1). Median retention time could not be calculated, as fewer than 50% of patients discontinued treatment during follow‐up. At last follow‐up, 89/108 patients (82.4%) had remained on CNB therapy.

### 3.4 Safety and tolerability

Adverse events (AE) occurred in 62 patients (57,4%) during treatment. The most common AE were fatigue (*n* = 54), vertigo (*n* = 19) and behavioral problems (*n* = 19). A rash was reported in 3 patients, but no Drug Reaction with Eosinophilia and Systemic Symptoms (DRESS) occurred. Retention differed by occurrence of documented treatment‐emergent AE (*p* = 0.0076). At 12 months, retention was 93.5% (95% CI: 86.6–100) in patients without AE and 81.6% (95% CI: 72.2–92.1) in those with AE (Figure S2). At the individual level, AE were the primary reason for treatment discontinuation in only five patients. Among these, two experienced a clinically relevant seizure reduction (>50%), while three showed no therapeutic benefit.

### 3.5 Efficacy outcomes

Estimated baseline seizure frequency was high, with a median of 30 seizures per month (IQR 91.2; range 1–750). At last follow‐up, the estimated median monthly seizure frequency was 9 (IQR 39.2; range 0–300) (*p* < 0.001, Wilcoxon test). (Figure 1A). Overall, 57/108 patients (52.8%) achieved ≥50% seizure reduction, including 20/108 (18.5%) who were seizure‐free during the documented interval between visits. An additional 17/108 (15.7%) had 1%–49% improvement, whereas 34/108 (31.5%) had no improvement or worsening (Figure 1B). In sensitivity analyses excluding patients with follow‐up ≤90 days and <180 days, ≥50% responder rates were 54.4% (56/103) and 56.1% (55/98), respectively. Baseline seizure frequency in the seizure‐free subgroup was lower than in the remaining cohort (median 7.5 vs. 31 seizures/month).

**FIGURE 1:**
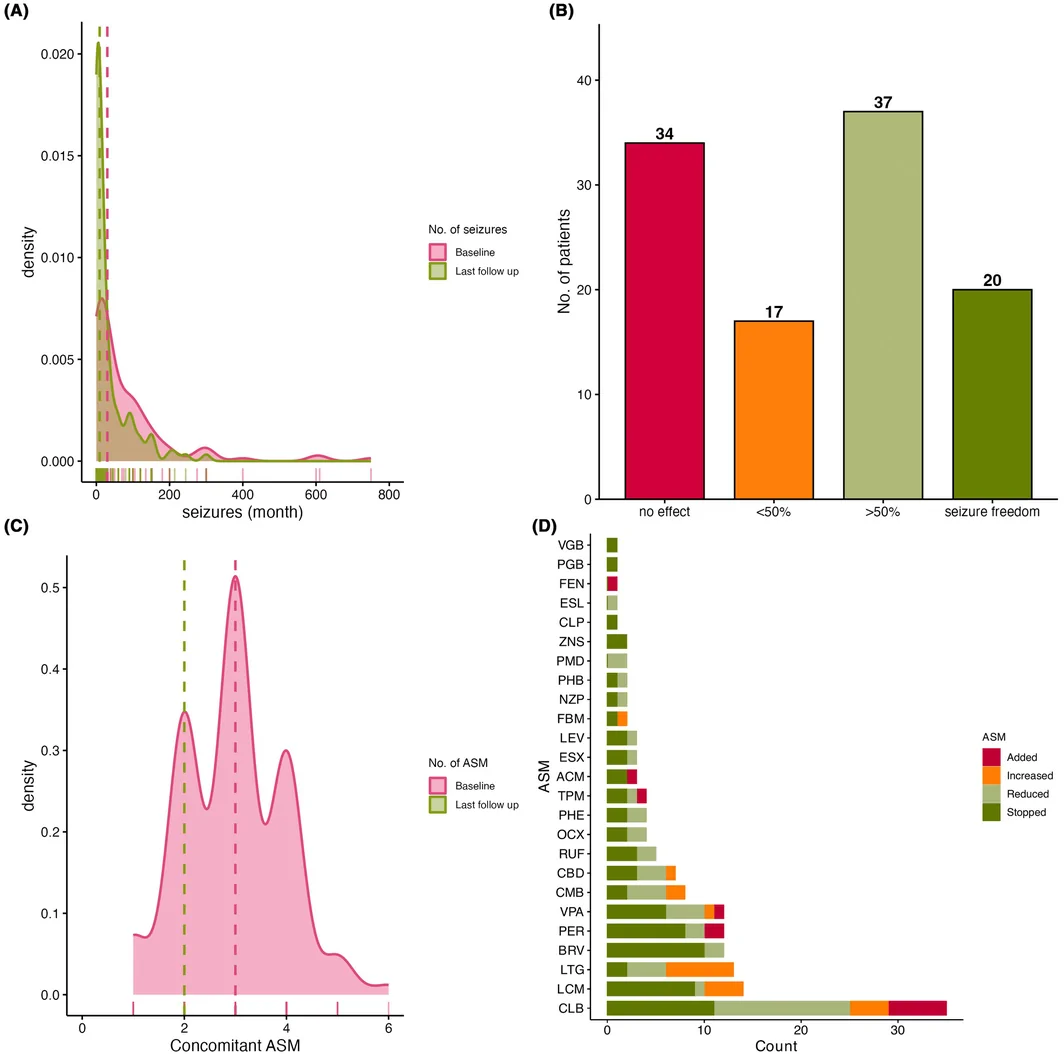
Outcome after off‐label treatment with cenobamate in pediatric drug‐resistant epilepsy. (A) CNB treatment resulted in a substantial reduction in monthly seizure frequency; the median declined from 30 seizures per month (IQR 91.25) at baseline to 9 (IQR 39.25) at last follow‐up. (B) Overall, 57/108 patients (52.8%) achieved a ≥50% seizure reduction, including 20/108 (18.5%) who were seizure‐free during the documented interval between visits; 17/108 (15.7%) had 1%–49% improvement, and 34/108 (31.5%) had no improvement or worsening. (C) The number of concomitant ASM significantly decreased during CNB treatment, with the median declining from 3 (IQR: 2) at baseline to 2 (IQR: 1) at last follow‐up. (D) Concomitant ASM regimens were frequently modified: 48 patients (44%) had at least one ASM dose‐reduced, and 55 patients (51%) discontinued one or more ASM entirely. New ASM were initiated in 11 patients (10%), and dose escalations occurred in 17 patients (16%). CLB showed the highest rate of discontinuation or dose reduction, with notable changes also observed for sodium channel–modulating agents like LCM and LTG. BRV, Brivaracetam; CBD, Cannabidiol; CI, Confidence Interval; CLB, Clobazam; CLP, Clonazepam; CMB, Cenobamate; CNB, Cenobamate; ESL, Eslicarbazepine; ESX, Ethosuximide; FBM, Felbamate; FEN, Fenfluramine; IQR, Interquartile Range; LCM, Lacosamide; LEV, Levetiracetam; LTG, Lamotrigine; NZP, Clonazepam; OCX, Oxcarbazepine; PER, Perampanel; PGB, Pregabalin; PHB, Phenobarbital; PHE, Phenytoin; PMD, Primidone; RUF, Rufinamide; TPM, Topiramate; VGB, Vigabatrin; VPA, Valproic Acid; ZNS, Zonisamide.

In exploratory univariable analyses, no significant baseline predictors of ≥50% response were identified, including structural etiology, genetic etiology, baseline ASM burden, baseline seizure frequency, or final weight‐adjusted CNB dose (all *p* > 0.15). Longer follow‐up was associated with response status (*p* = 0.012), underscoring the importance of follow‐up heterogeneity. Patients achieving seizure freedom received the lowest median weight‐adjusted dose (2.72 mg/kg/day), compared to those with partial response (3.73 mg/kg/day), non‐response (2.99 mg/kg/day), or <50% improvement (3.73 mg/kg/day) (Figure S3).

### 3.6 Concomitant ASM

During the treatment period, modifications to concomitant ASM regimens were frequent. In 48/108 patients (44.4%), at least one ASM dose was reduced, and 55/108 (50.9%) discontinued one or more ASM entirely. In contrast, new ASM were initiated in 11/108 (10.2%), and dose escalations occurred in 17/108 (15.7%). (Figure 1C). Overall, the median number of concomitant ASM declined from 3 (IQR 2; range 1–6) at baseline to 2 (IQR 1; range 0–5) at last follow‐up. Clobazam showed the highest rate of dose reduction or discontinuation, and notable changes were also observed for lacosamide and lamotrigine (Figure 1D). These regimen modifications may have reflected improved seizure control, tolerability, or management of pharmacodynamic and pharmacokinetic interactions.

## 4 DISCUSSION

In this large European cohort of 108 pediatric DRE patients, adjunctive cenobamate achieved meaningful seizure control. Overall, 52.8% achieved ≥50% seizure reduction, including 18.5% who were seizure‐free during the documented interval between visits. Responder rates remained similar after exclusion of patients with follow‐up ≤90 days and <180 days.

While seizure freedom rates in our cohort are comparable to those reported in a recent meta‐analysis, the proportion of patients achieving significant seizure reduction appears to be lower.^6^ This likely reflects the high refractoriness of our cohort, with many having received multiple ASM and other non‐medical therapies such as ketogenic diet, Vagus nerve stimulation, or resective epilepsy surgery. Despite this, the observed outcomes remain clinically meaningful. However, these efficacy estimates must be interpreted cautiously because seizure counts were retrospectively abstracted from routine chart documentation rather than standardized prospective seizure diaries, and seizure freedom referred to the interval between visits rather than sustained long‐term seizure freedom. The lower baseline seizure burden in the seizure‐free subgroup (median 7.5 vs. 31 seizures/month) warrants caution when comparing these outcomes with studies using more stringent 6‐ or 12‐month seizure‐freedom definitions. Likewise, no clear baseline clinical predictor of response emerged in univariable analyses. Within these constraints, our findings are broadly consistent with previous adult and smaller pediatric real‐world reports. Differences may also stem from the broader, multicenter composition of our cohort. Real‐world adult studies also report robust efficacy.^9^ Further small case series also show its efficacy in developmental and epileptic encephalopathies and Lennox Gastaut Syndrome.^10^, ^11^ Due to the retrospective design and heterogeneity of underlying etiologies, further subgroup analysis by etiology or ILAE classification was not feasible.

Cenobamate was generally well tolerated in our pediatric cohort. AE (57% of patients) were mostly mild (predominantly fatigue/somnolence), consistent with other reports.^7^ Similarly, ~75% of adults in a recent series reported AEs (mostly fatigue and somnolence).^8^ Common adverse effects mirror the pivotal trials.^12^ However, AEs were retrospectively extracted from charts, were not formally graded, and were probably underascertained because they were not actively solicited across centers. Accordingly, the observed AE profile should be interpreted as a minimum documented burden rather than a comprehensive safety estimate.

Notably, more than half our patients reduced or discontinued concomitant ASM during cenobamate therapy, particularly clobazam and sodium channel–modulating agents such as lacosamide and lamotrigine. These changes should not be interpreted solely as a consequence of seizure improvement because cenobamate has clinically relevant drug–drug interaction potential and some dose reductions or discontinuations may have been undertaken for safety or tolerability reasons.

Cenobamate retention at 12 months was high in our cohort (86.8%), indicating sustained clinical benefit in most patients. Retention was notably higher among those without adverse events (93.5% vs. 81.6%), consistent with observations in adult cohorts, where tolerability is a key factor influencing treatment continuation.^13^ These results compare favorably with adult data, where pooled trial estimates suggest a 12‐month retention rate of approximately 80%, and recent real‐world studies report rates around 82%.^13^, ^14^ The high retention observed in our pediatric DRE population reinforces the impression that, once initiated and tolerated, cenobamate is generally maintained as part of long‐term therapy.

This retrospective study also has additional limitations. Data collection and follow‐up were heterogeneous across centers, and only centers with prior pediatric CNB experience contributed patients, introducing potential selection bias. Missing data were handled by available‐case analysis without imputation. Dosing and titration were off‐label and not standardized, reflecting real‐world practice. The lack of a control group precludes causal inferences regarding efficacy or safety. More detailed subclassification of the genetic etiology group was not consistently available across centers, and developmental or cognitive outcomes could not be assessed.

## 5 CONCLUSION

Cenobamate showed clinically meaningful efficacy and was generally well tolerated in this multicenter retrospective cohort of pediatric patients with drug‐resistant epilepsy. Overall, 52.8% achieved ≥50% seizure reduction, including 18.5% who were seizure‐free during the documented interval between visits. Adverse events were common but mostly mild, and the 12‐month retention rate exceeded 85%, reflecting good treatment adherence. Because efficacy and safety were derived from heterogeneous chart‐based follow‐up without standardized seizure diaries or formal AE grading, these findings should be interpreted cautiously. Prospective controlled studies are needed to define sustained seizure‐freedom rates, tolerability, interaction management, and optimal patient selection in children.

## AUTHOR CONTRIBUTIONS

Konstantin L. Makridis: Conceptualization, methodology, investigation, formal analysis, writing – original draft, writing – review and editing, visualization. Thomas Bast: Investigation, writing – review and editing. Stéphane Auvin: Investigation, writing – review and editing. Pavel Krsek: Investigation, writing – review and editing. Matyas Ebel: Investigation, writing – review and editing. Eva Breuer: Investigation, writing – review and editing. Mira Beckhaus: Investigation, writing – review and editing. Matúš Bašovský: Investigation, writing – review and editing. Pavlína Danhofer: Investigation, writing – review and editing. Ondřej Horák: Investigation, writing – review and editing. Alessandro Orsini. Investigation, writing – review and editing. Gonzalo Alonso Ramos Rivera. Investigation, writing – review and editing. Hana Medřická: Investigation, writing – review and editing. Steffen Syrbe: Investigation, writing – review and editing. Angela M. Kaindl: Investigation, methodology, writing – review and editing; supervision.

## FUNDING INFORMATION

The study was supported by the Einstein Stiftung Fellowship through the Günter Endres Fond, the Sonnenfeld‐Stiftung, and the Federal Ministry of Education and Research (Bundesministerium für Bildung und Forschung, BMBF) as part of the German Center for Child and Adolescent Health (DZKJ).

## CONFLICT OF INTEREST STATEMENT

K.L.M. served as a speaker and received travel expenses from Angelini Pharma and Jazz Pharmaceuticals. A.M.K. has nothing to report. We confirm that we have read the Journal's position on issues involved in ethical publication and affirm that this report is consistent with those guidelines.

## ETHICAL STATEMENT

We confirm that we have read the Journal's position on issues involved in ethical publication and affirm that this report is consistent with those guidelines.

## Supporting information


**Figure S1** Retention analyses for off‐label cenobamate treatment in pediatric drug‐resistant epilepsy. Kaplan–Meier estimated probability of overall treatment retention on cenobamate (CNB), shown with numbers at risk over time. Estimated retention was 91.7% (95% CI 86.6–97.0) at 6 months and 86.8% (95% CI 80.5–93.5) at 12 months.

**Figure S2** Retention analyses for off‐label cenobamate treatment in pediatric drug‐resistant epilepsy. Kaplan–Meier estimated probability of treatment retention stratified by documented treatment‐emergent adverse events, shown with numbers at risk over time. At 12 months, retention was 93.5% (95% CI 86.6–100) in patients without documented adverse events and 81.6% (95% CI 72.2–92.1) in those with documented adverse events (log‐rank *p* = 0.0076).

**Figure S3** Violin plot showing final weight‐adjusted cenobamate dose (mg/kg/day) stratified by seizure outcome category. In exploratory subgroup analyses, one‐way ANOVA revealed no statistically significant differences in dose across outcome groups (*F* [3103] = 1.53, *p* = 0.21, generalized eta squared = 0.043). Post hoc comparisons using Tukey's test showed no significant pairwise differences (all *p* > 0.25).

## Data Availability

The data that support the findings of this study are available from the corresponding author upon reasonable request.
