# Supplementary figures and images for: Real‐world use of Cenobamate in pediatric drug‐resistant epilepsy: A European multicenter retrospective study
Source: Epilepsia Open. 2026 Jul 3:10.1002/epi4.70301. Online ahead of print. doi: 10.1002/epi4.70301 (PMC13394996; doi:10.1002/epi4.70301)

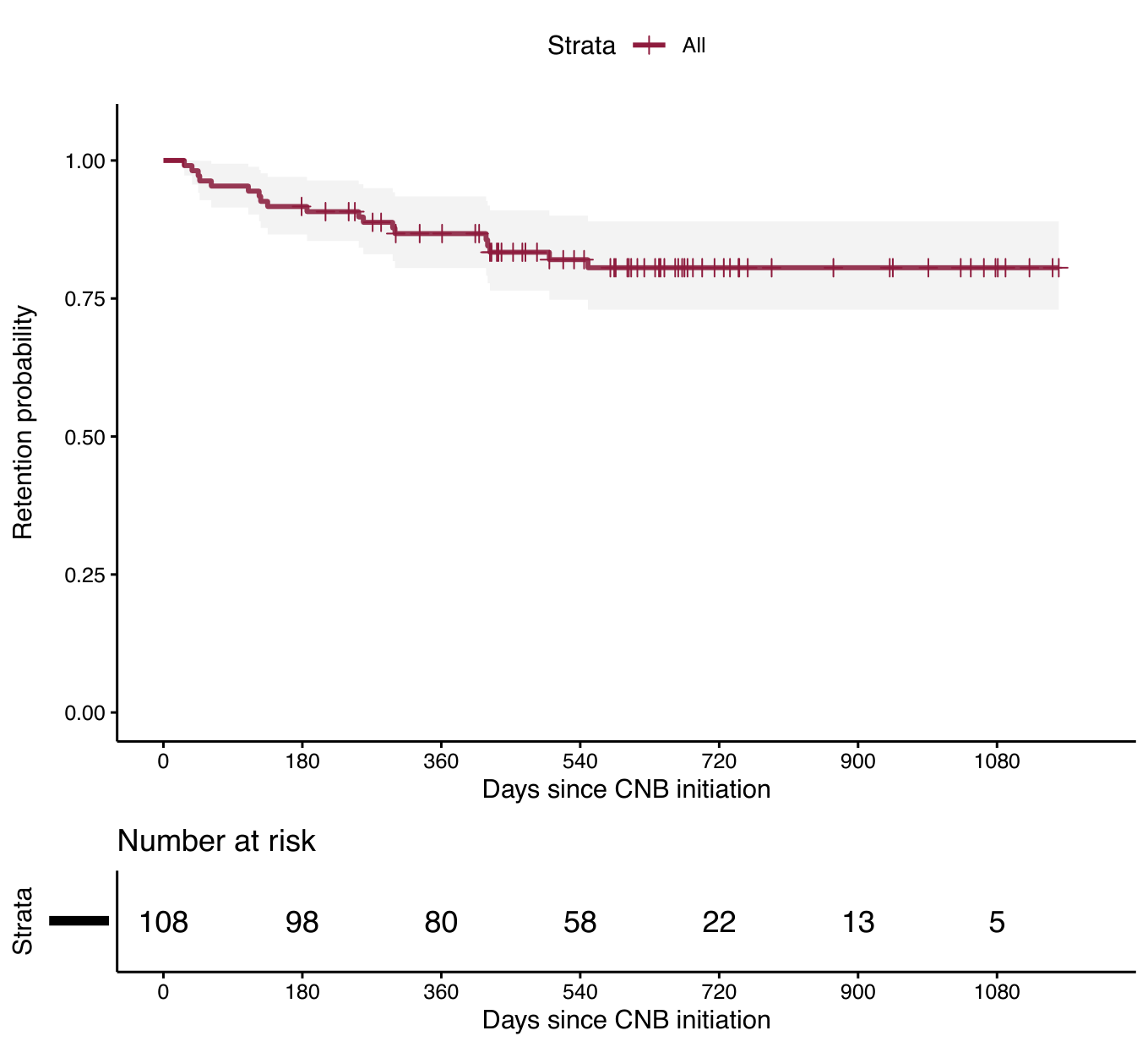

Supplement: Supplementary file 1 — Figure S1 Retention analyses for off‐label cenobamate treatment in pediatric drug‐resistant epilepsy. Kaplan–Meier estimated probability of overall treatment retention on cenobamate (CNB), shown with numbers at risk over time. Estimated retention was 91.7% (95% CI 86.6–97.0) at 6 months and 86.8% (95% CI 80.5–93.5) at 12 months. [file EPI4-9999-0-s001.tiff]

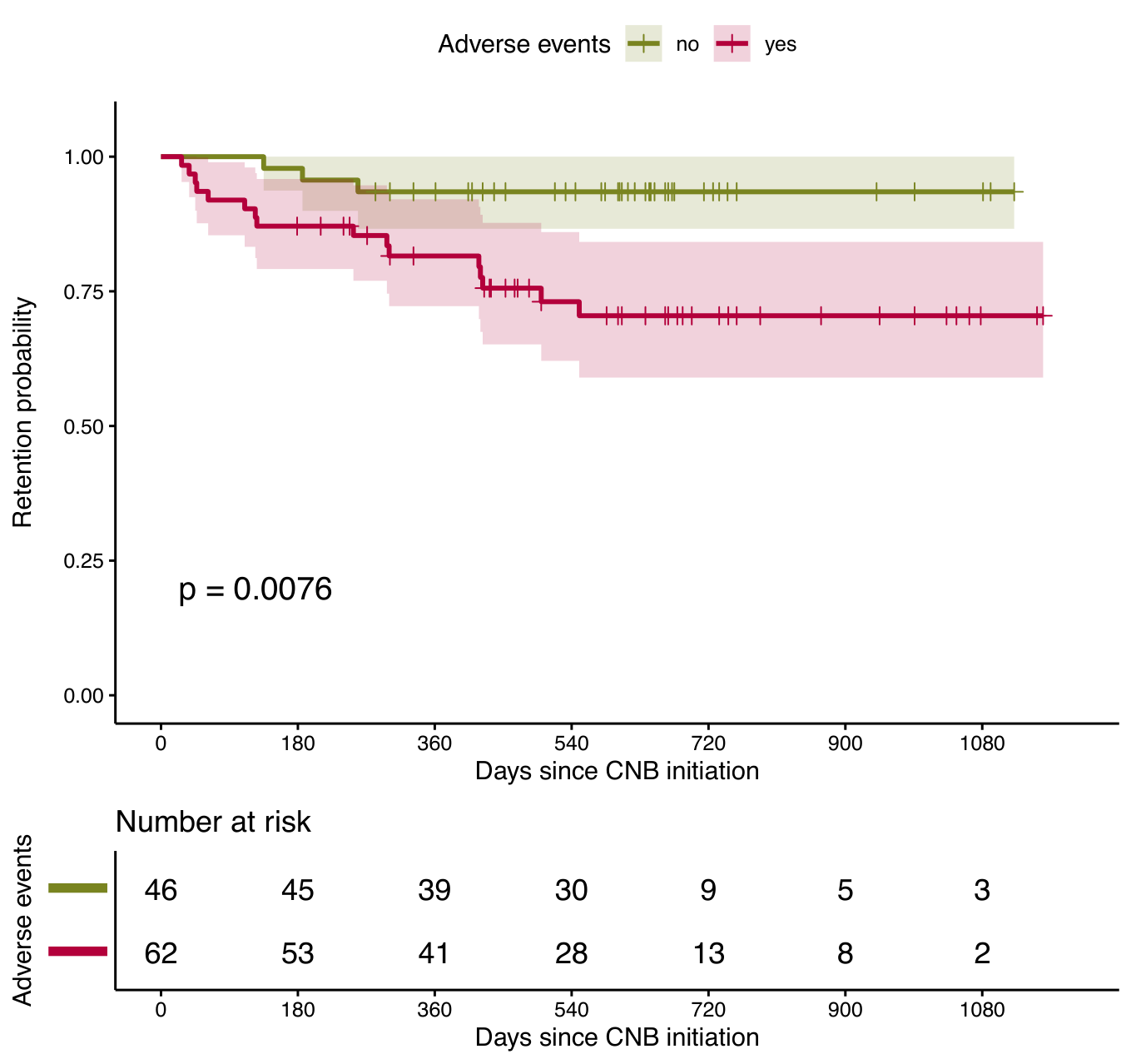

Supplement: Supplementary file 2 — Figure S2 Retention analyses for off‐label cenobamate treatment in pediatric drug‐resistant epilepsy. Kaplan–Meier estimated probability of treatment retention stratified by documented treatment‐emergent adverse events, shown with numbers at risk over time. At 12 months, retention was 93.5% (95% CI 86.6–100) in patients without documented adverse events and 81.6% (95% CI 72.2–92.1) in those with documented adverse events (log‐rank p = 0.0076). [file EPI4-9999-0-s003.tiff]

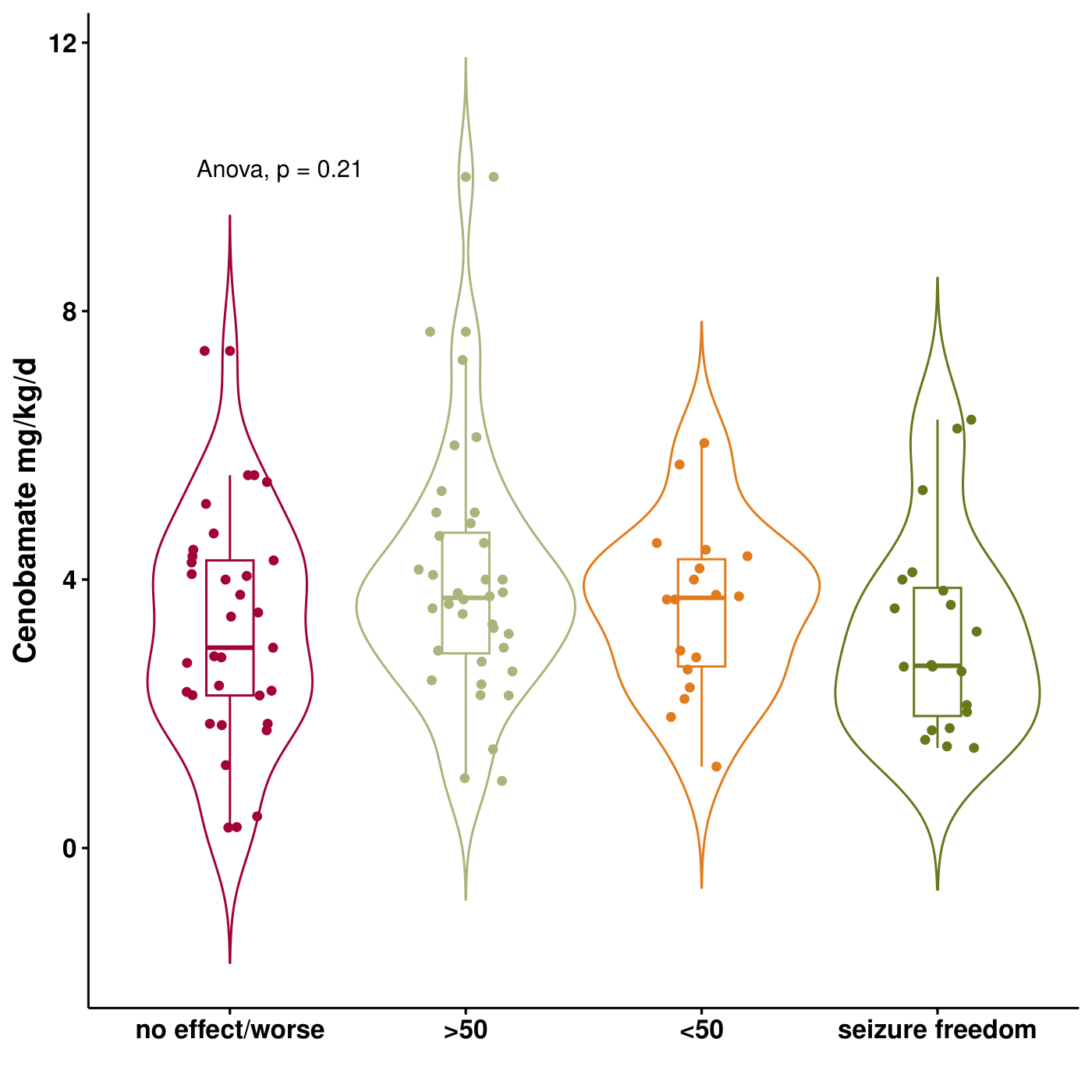

Supplement: Supplementary file 3 — Figure S3 Violin plot showing final weight‐adjusted cenobamate dose (mg/kg/day) stratified by seizure outcome category. In exploratory subgroup analyses, one‐way ANOVA revealed no statistically significant differences in dose across outcome groups (F [3103] = 1.53, p = 0.21, generalized eta squared = 0.043). Post hoc comparisons using Tukey's test showed no significant pairwise differences (all p > 0.25). [file EPI4-9999-0-s002.tiff]
